# The Transcription Factor GLI1 Mediates TGFβ1 Driven EMT in Hepatocellular Carcinoma via a SNAI1-Dependent Mechanism

**DOI:** 10.1371/journal.pone.0049581

**Published:** 2012-11-19

**Authors:** Xin Zheng, Natalia B. Rumie Vittar, Xiaohong Gai, Maite G. Fernandez-Barrena, Catherine D. Moser, Chunling Hu, Luciana L. Almada, Angela L. McCleary-Wheeler, Sherine F. Elsawa, Anne M. Vrabel, Abdirashid M. Shire, Andrea Comba, Snorri S. Thorgeirsson, Youngsoo Kim, Qingguang Liu, Martin E. Fernandez-Zapico, Lewis R. Roberts

**Affiliations:** 1 Department of Hepatobiliary Surgery, The First Affiliated Hospital of Xi'an Jiaotong University, Xi'an, Shaanxi, China; 2 Division of Gastroenterology and Hepatology, Mayo Clinic, Rochester, Minnesota, United States of America; 3 Mayo Clinic Cancer Center, Mayo Clinic, Rochester, Minnesota, United States of America; 4 Schulze Center for Novel Therapeutics, Mayo Clinic, Rochester, Minnesota, United States of America; 5 Department of Biological Sciences, Northern Illinois University, DeKalb, Illinois, United States of America; 6 Laboratory of Experimental Carcinogenesis, Center for Cancer Research, National Cancer Institute, National Institutes of Health, Bethesda, Maryland, United States of America; 7 Isis Pharmaceuticals Inc., Carlsbad, California, United States of America; National Cancer Center, Japan

## Abstract

The role of the epithelial-to-mesenchymal transition (EMT) during hepatocellular carcinoma (HCC) progression is well established, however the regulatory mechanisms modulating this phenomenon remain unclear. Here, we demonstrate that transcription factor glioma-associated oncogene 1 (GLI1) modulates EMT through direct up-regulation of SNAI1 and serves as a downstream effector of the transforming growth factor-β1 (TGFβ1) pathway, a well-known regulator of EMT in cancer cells. Overexpression of GLI1 increased proliferation, viability, migration, invasion, and colony formation by HCC cells. Conversely, GLI1 knockdown led to a decrease in all the above-mentioned cancer-associated phenotypes in HCC cells. Further analysis of GLI1 regulated cellular functions showed that this transcription factor is able to induce EMT and identified SNAI1 as a transcriptional target of GLI1 mediating this cellular effect in HCC cells. Moreover, we demonstrated that an intact GLI1-SNAI1 axis is required by TGFβ1 to induce EMT in these cells. Together, these findings define a novel cellular mechanism regulated by GLI1, which controls the growth and EMT phenotype in HCC.

## Introduction

HCC is the third most frequent cause of cancer death, with an estimated 750,000 new cases per year [1]. Liver resection and transplantation are currently the main curative therapies for HCC [2], [3]. Unfortunately, there is a high rate of postsurgical recurrence after resection due to metastatic dissemination of the tumor prior to resection or to the development of new neoplastic changes in the remaining cirrhotic liver [4], [5]. Consequently, the long-term prognosis of most patients with HCC is extremely poor [6], [7]. Hence, it is critical to improve our understanding of the molecular mechanisms determining HCC recurrence and metastasis to develop new therapeutic strategies for this disease.

The transition of epithelial cells to a mesenchymal phenotype, which is designated as epithelial-to-mesenchymal transition (EMT), has been increasingly recognized to occur during the progression of various carcinomas, including HCC [8], [9]. It has been proposed that EMT is one of the key mechanisms through which metastasis occurs in different tumors, beginning with the disruption of intercellular contacts and the enhancement of cell motility, thus resulting in the release of cancer cells from the primary tumor. Several studies have shown that different pathways are capable of inducing the EMT phenotype in HCC cells [8], [10]–[12], however, the specific mechanisms regulating this phenomenon are still incompletely understood. We have previously reported that the expression of GLI1, a well-known oncogenic transcription factor, in HCC tissues was positively associated with the EMT phenotype [11]. Here, we expanded on these findings and investigated the functional role of GLI1 in HCC and the interaction between GLI1, TGFβ1 and SNAI1 in the context of the EMT, and finally defined novel molecular events underlying the EMT in HCC. These findings further support the role of the transcription factor GLI1 in the regulation of EMT and expand the repertoire of molecules including ZEB1, ZEB2, SNAI2 and TWIST [13]–[15] that act in concert with TGFβ1 and GLI1 pathways to control EMT in cancer cells.

## Results

### GLI1 enhances HCC colony formation and promotes cell proliferation, viability, migration and invasion

To develop *in vitro* models xamining the mechanistic role of GLI1 in HCC biology, we determined the mRNA expression of GLI1 in 11 different human HCC cell lines and normal human hepatocytes by qRT-PCR. Five of the HCC cell lines (PLC/PRF5, SNU182, SNU398, SNU449, and SNU475) express GLI1 mRNA at a higher level than normal human hepatocytes, with four of the cell lines expressing GLI1 at more than two-fold the level in normal hepatocytes (Figure S1). SNU398, the highest expressing cell line, expressed GLI1 mRNA at over 55-times the level in normal hepatocytes. Cell lines expressing lower GLI1 mRNA include Hep3B, HepG2, Huh7, SK-HEP-1, SNU387 and SNU423 (Figure S1). We confirmed that GLI1 protein expression mirrors the mRNA expression in a subset of HCC cell lines using Western blot analysis. Similar to the data shown in Figure S1, SNU398 shows the highest GLI1 protein expression and Huh7 is among the HCC cells with the lowest GLI1 expression (Figure S2). We selected Huh7 for GLI1 overexpression experiments; and SNU398 cells to be transfected with GLI1 antisense oligonucleotides (ASO) in knockdown experiments.

Initially, to investigate the effect of GLI1 on HCC cell growth, we overexpressed a FLAG-tagged expression construct of this transcription factor. Overexpression of GLI1 was confirmed by qRT-PCR and Western immunoblotting. Western immunoblotting and RT-PCR confirmed that Huh-7 cells transfected with GLI1 (Huh7-GLI1) cells expressed GLI1 protein at a high level compared to the Huh7 cells transfected with parental vector (Huh7-Vector) (Figure S3A). Similarly, GLI-dependent reporter activity was increased after transfection of Huh7 and SNU423 cells with GLI1, confirming the functional capacity of the expressed protein (*P*<0.0001; Figure S3B and data not shown).

**Figure 1:**
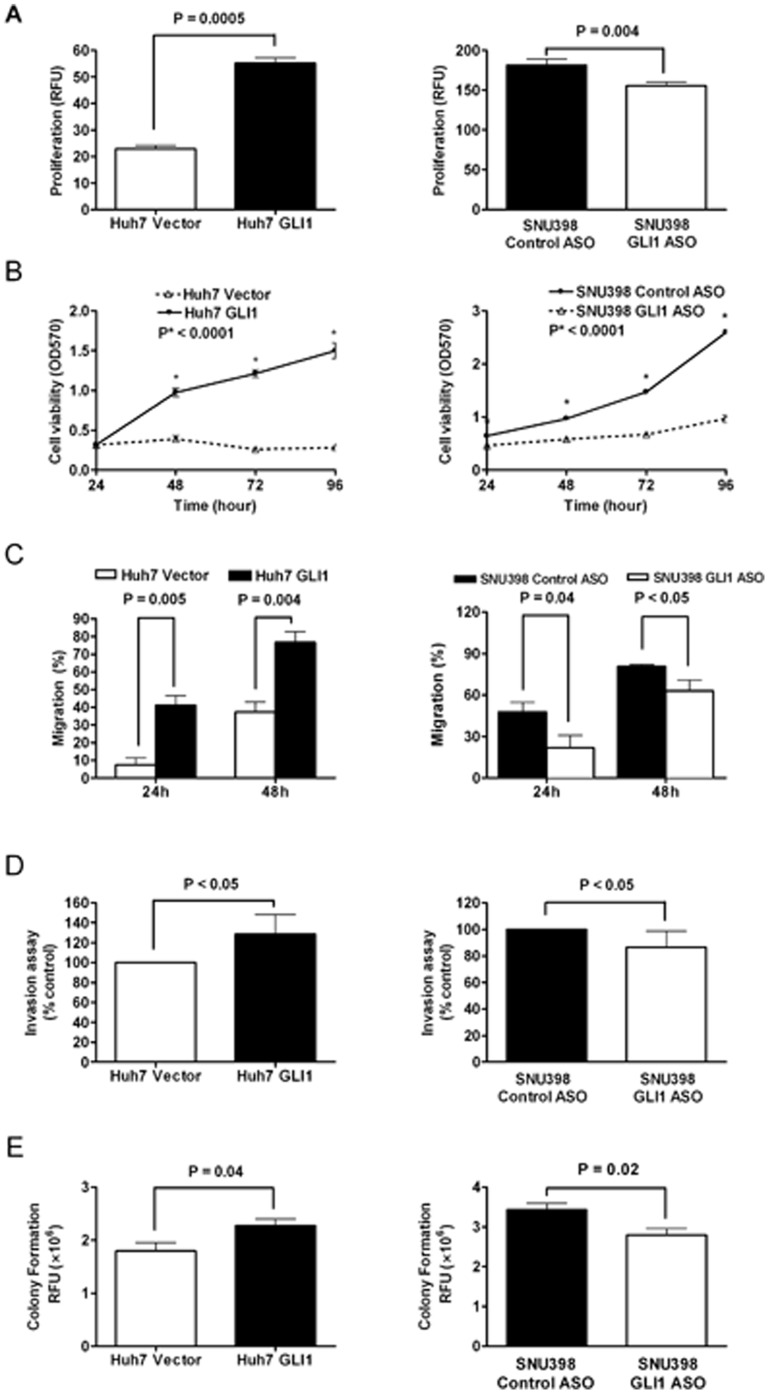
GLI1 promotes cell proliferation, viability, migration, invasion and colony formation of HCC cells. (A) Overexpression of GLI1 increases BrdU incorporation of Huh7 cells and silencing GLI1 expression in SNU398 cells decreases BrdU incorporation. (B) As assessed by the MTT assay, cell viability is increased at 48, 72 and 96 hours by overexpression of GLI1 in Huh7 cells and decreased at 24, 48, 72 and 96 hours by knockdown of GLI1 in SNU398 cells. (C) As assessed by the wound-healing assay, at both 24 and 48 hours, cell migration rate is increased in Huh7 cells by overexpression of GLI1 and decreased in SNU398 cells by silencing GLI1 expression. (D) Cell invasion is increased by overexpression of GLI1 in Huh7 cells and decreased by knockdown of GLI1 in SNU398 cells. (E) Overexpression of GLI1 promotes colony formation of Huh7 cells; in contrast, knockdown of GLI1 represses colony formation of SNU398 cells.

Next, we quantitated cell proliferation using the BrdU incorporation assay and cell viability using the MTT assay. Huh7-GLI1 cells showed significantly greater BrdU incorporation than Huh7-Vector cells (*P* = 0.0005; Figure 1A). Overexpression of GLI1 also significantly increased the viability of Huh7 cells as assessed by the MTT assay (*P*<0.0001 at all four time points; Figure 1B). To determine whether ectopic expression of GLI1 increases HCC cell migration, we used the wound-healing assay and showed that the migration rate of Huh7-GLI1 cells was significantly higher than that of Huh7 Vector cells at both 24 and 48 hours after scratching (*P* = 0.005 and *P* = 0.004, respectively; Figure 1C). To determine the effect of GLI1 on HCC invasion, we performed a quantitative transwell chamber invasion assay. Overexpression of GLI1 significantly increased the invasive ability of Huh7 cells (*P*<0.05; Figure 1D). Further characterization of the cellular functions regulated by GLI1 using the soft agar colony formation assay showed there were significantly more cell colonies formed by Huh7 cells stably-expressing GLI1 (*P* = 0.04; Figure 1E).

To complement these overexpression studies, we evaluated the effect of suppression of GLI1 on the cancer phenotype by transfecting the endogenously high GLI1-expressing SNU398 cells with GLI1 ASO. Suppression of GLI1 mRNA expression was confirmed by qRT-PCR 24 hours after transfection. GLI1 mRNA was decreased about 90% after transfection with GLI1 ASO (*P* = 0.002; Figure S3A). Western immunoblotting confirmed the decrease in GLI1 protein expression in SNU398-GLI1 ASO cells (Figure S3A). A GLI reporter luciferase assay was used to confirm the functional consequence of transfection with GLI1 ASO. Luciferase activity decreased significantly in SNU398 GLI1 ASO transfected cells compared to control ASO transfected cells (*P* = 0.002; Figure S3B and data not shown). Cell proliferation as measured by the BrdU ELISA assay (*P* = 0.004; Figure 1A), cell viability as measured by the MTT assay (*P*<0.0001 at all four time points; Figure 1B), cell migration (*P*<0.05 and *P*<0.05, respectively at 24 and 48 hrs; Figure 1C), and cell invasion (*P*<0.05; Figure 1D) were all significantly decreased in SNU398-GLI1 ASO cells as compared to SNU398-Control ASO cells. Similarly, the soft agar colony formation assay showed that fewer colonies were formed by SNU398-GLI1 ASO transfected cells compared to the control cells (*P* = 0.04; Figure 1E).

**Figure 2:**
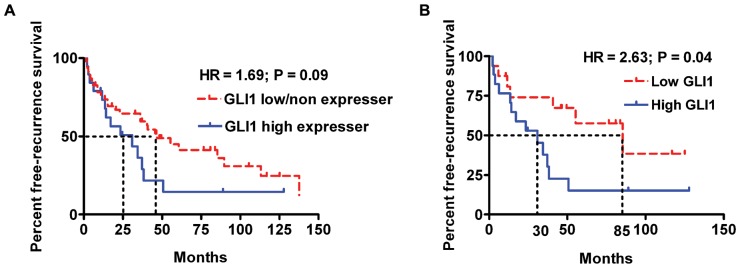
GLI1 expression is positively associated with HCC recurrence. (A) Tumor recurrence after surgical resection in 139 patients with HCCs. Differences between the Kaplan-Meier curves of patients in the GLI1 high expresser group and those in GLI1 low/non expresser group using the log rank test. (B) Tumor recurrence after surgical resection in the 35 patients from the high GLI1 group and in the 35 patients from the low GLI1 group. Increased GLI1 expression in HCC tissues was significantly correlated with more rapid tumor recurrence after surgical resection of the primary tumor.

To determine the clinical relevance of the above-mentioned effects of GLI1 in HCC cells, we examined the association between GLI1 mRNA expression and HCC recurrence by gene expression microarray. GLI1 mRNA was detected in 74 of 139 HCC patients and there was increased GLI1 expression in tumor compared to benign tissues in 43 (58.1%) of the HCCs. The 139 HCC patients were classified into two groups: GLI1 high expresser and GLI1 low/non expresser. The GLI1 high expresser group included patients with higher GLI1 expression in tumor tissues, while the GLI1 low/non expresser group included patients with lower or no GLI1 expression in tumor tissues. As shown in Table S1, there were no significant differences in demographic and clinical characteristics between these two groups except for vascular invasion, which was more prevalent in high GLI1 expressing tumors (*P* = 0.05). Although there was no significant difference in overall survival time between the two groups, the 5-year recurrence rate in the GLI1 high expresser group was 85% compared to 59% in the GLI1 low/non expresser group (*P* = 0.007, Chi square test), and comparison of Kaplan Meier recurrence-free survival curves showed a tendency toward a more rapid rate of tumor recurrence in patients with GLI1 high expressing tumors (HR = 1.69; 95% CI: 0.9, 3.9; *P* = 0.09; Figure 2A). The median recurrence-free survival was 25 months in the GLI1 high expresser group, while the median recurrence-free survival in the GLI1 low/non expresser group was 47 months (Figure 2A). When patients were classified into two groups by GLI1 expression, a Low GLI1 and High GLI1 group using the median ratio of tumor/benign GLI1 expression as the cut-off value, most demographic and clinical characteristics were similar in the two groups, except that there were more HCC patients with alcoholic liver disease in the High GLI1 group (*P*<0.05) (Table S2). Consistent with the prior results, tumor vascular invasion was more frequent in High GLI1 expressing HCCs than in Low GLI1 expressing HCCs (22.9% vs. 14.3%), but this difference was not statistically significant (Table S2). The 5-year recurrence rate in the High GLI1 group was 85% compared to 42% in the Low GLI1 group. Comparison of the Kaplan Meier curves of recurrence-free survival showed a significantly more rapid rate of tumor recurrence in patients with tumors expressing high levels of GLI1 (HR = 2.63; 95% CI: 1.0, 6.5; *P* = 0.04; Median recurrence-free survival was 30 months in the High GLI1 group vs. 85 months in the Low GLI1 group; Figure 2B). Together these findings support a novel role for GLI1 in HCC pathogenesis and suggest tumor recurrence as one of the biological features that may be modulated by this transcription factor.

### GLI1 induces EMT in HCC cells through a SNAI1-dependent mechanism

**Figure 3:**
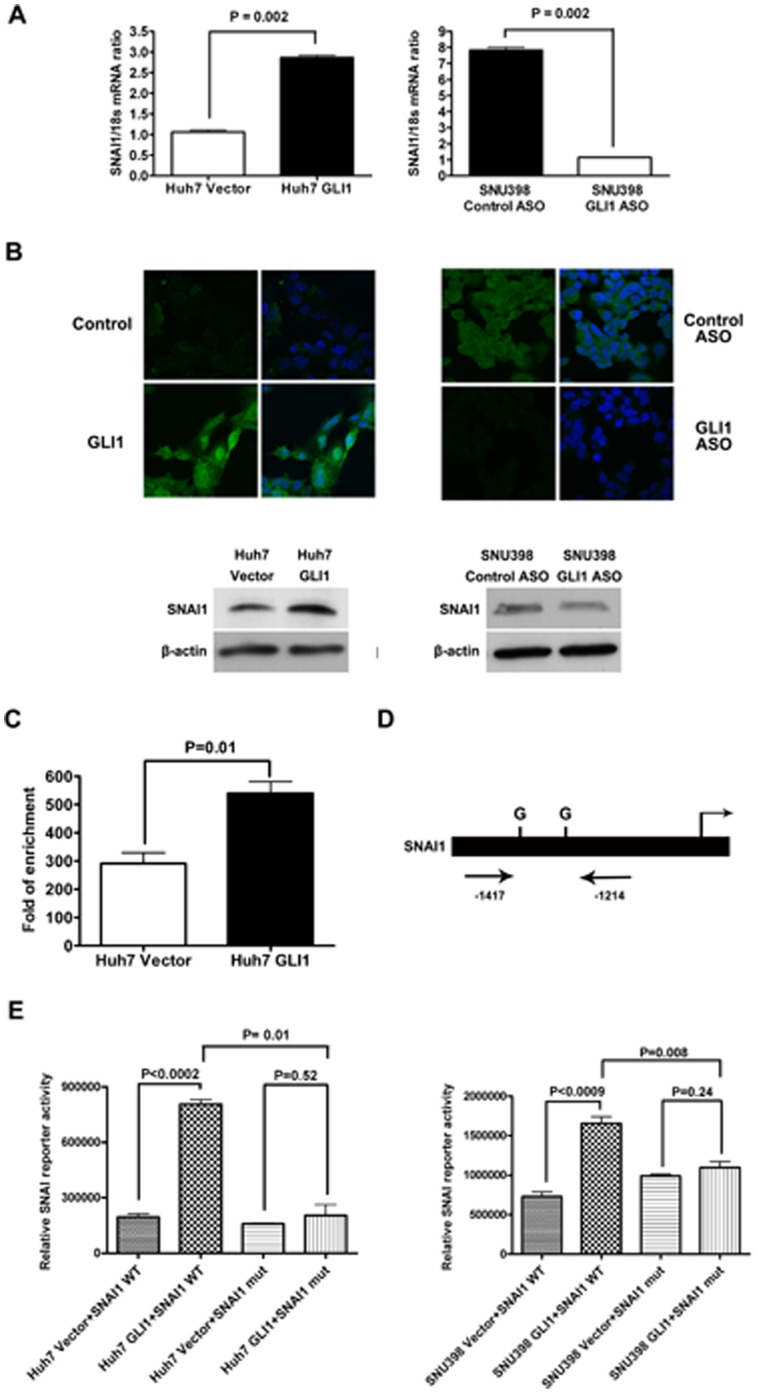
SNAI1 is a transcriptional target of GLI1 in HCC cells. (A) As measured by both qRT-PCR, SNAI1 expression is increased by overexpression of GLI1 in Huh7 cells and decreased by knockdown of GLI1 in SNU398 cells. (B) Western immunoblotting (lower panel) and immunofluorescence staining (upper panel) confirm that SNAI1 protein expression is increased by overexpression of GLI1 in Huh7 cells (left panel) and decreased by knockdown of GLI1 in SNU398 cells (right panel). (C) ChIP assay verifies that GLI1 protein binds to the SNAI1 promoter. PCR was performed using a specific set of primers as indicated in Methods. (D) Bioinformatics analysis of the SNAI1 promoter identified candidate GLI (G) binding sites; (TSS = transcription start site). The positive amplicon for the ChIP assay is marked −1,417/−1,214 bp upstream of transcription start site (E) Relative changes in luciferase activity in Huh7 (left) and SNU398 (right) cells transfected with the WT and GLI mutant SNAI1 promoter luciferase reporter and control vector or GLI1.

Analysis of the mechanism underlying the effect of GLI1 on HCC cell growth showed that SNAI1, a target of this transcription factor [16], [17] was significantly increased in Huh7 cells overexpressing GLI1 at the mRNA (*P* = 0.0022; Figure 3A) and protein levels, as confirmed by immunofluorescence (Figure 3B, upper right panel) and Western immunoblotting (Figure 3B, lower right panel). Consistent with the results in Huh7 cells, knockdown of GLI1 in SNU398 cells significantly reduced SNAI1 mRNA expression by about 85% (*P* = 0.002; Figure 3A, left panel) and also decreased SNAI1 protein levels (Figure 3B, left panel). Knockdown of GLI1 also reduced SNAI1 expression in SNU449 and SNU475 HCC cells (Figure S4). Finally, chromatin immunoprecipitation (ChIP) assays carried out in Huh7 cells showed that GLI1 can bind to a region in the SNAI1 promoter located −1,417/−1,214 bp upstream of the transcriptional start site (Figure 3C). Bioinformatics analysis of this region in the SNAI1 promoter demonstrates the presence of two candidate GLI1 bindings sites in this area (Figure 3D). Functional validation of these sites using luciferase reporter assays shows that GLI1 is not longer able to induce the activity of the SNAI1 promoter in a reporter construct lacking these two GLI1 bindings sites (Figure 3E). These data strongly suggest that GLI1 directly up-regulates SNAI1 expression in HCC and define SNAI1 as a direct target of the GLI1 transcription factor in HCC cells.

**Figure 4:**
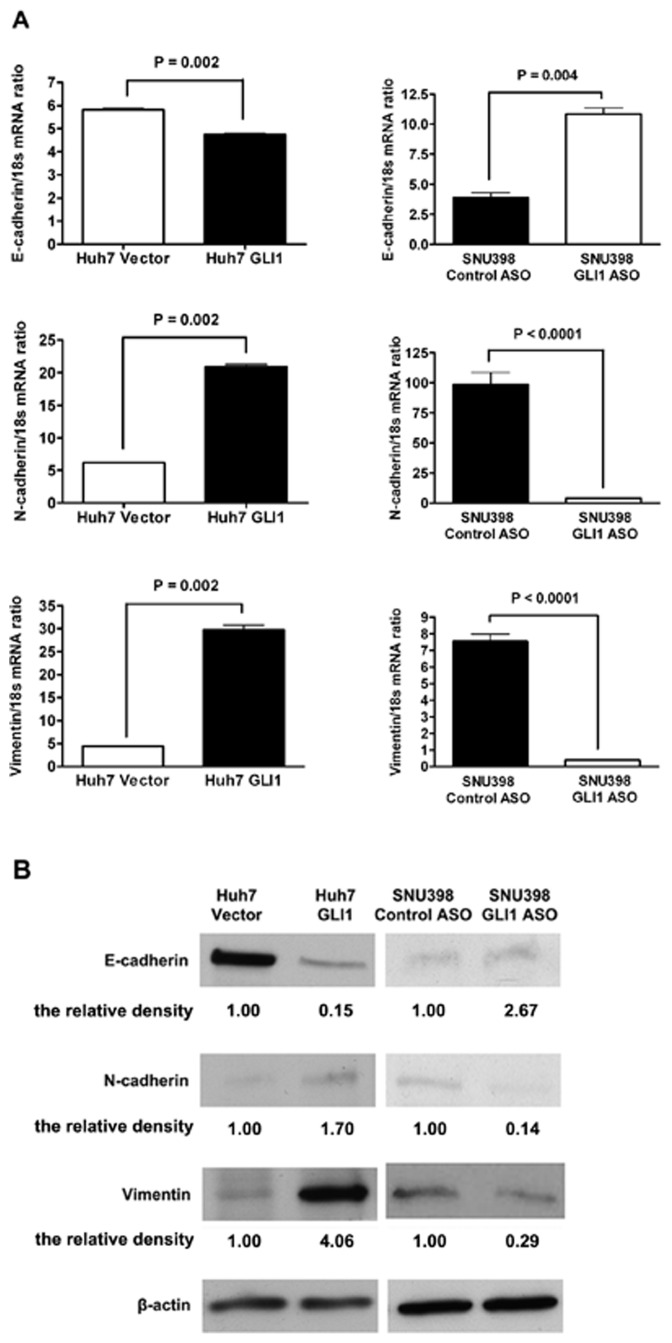
GLI1 induces EMT in HCC ells. (A) As assessed by qRT-PCR, overexpression of GLI1 decreases E-cadherin mRNA expression and increases the mRNA expression of both N-cadherin and Vimentin in Huh7 cells, while knockdown of GLI1 increases E-cadherin mRNA expression and decreases the mRNA expression of both N-cadherin and Vimentin in SNU398 cells. (B) As assessed by Western immunoblotting followed by densitometry analysis, overexpression of GLI1 decreases E-cadherin expression and increases the expression of both N-cadherin and Vimentin in Huh7 cells, while knockdown of GLI1 increases E-cadherin expression and decreases the expression of both N-cadherin and Vimentin in SNU398 cells.

**Figure 5:**
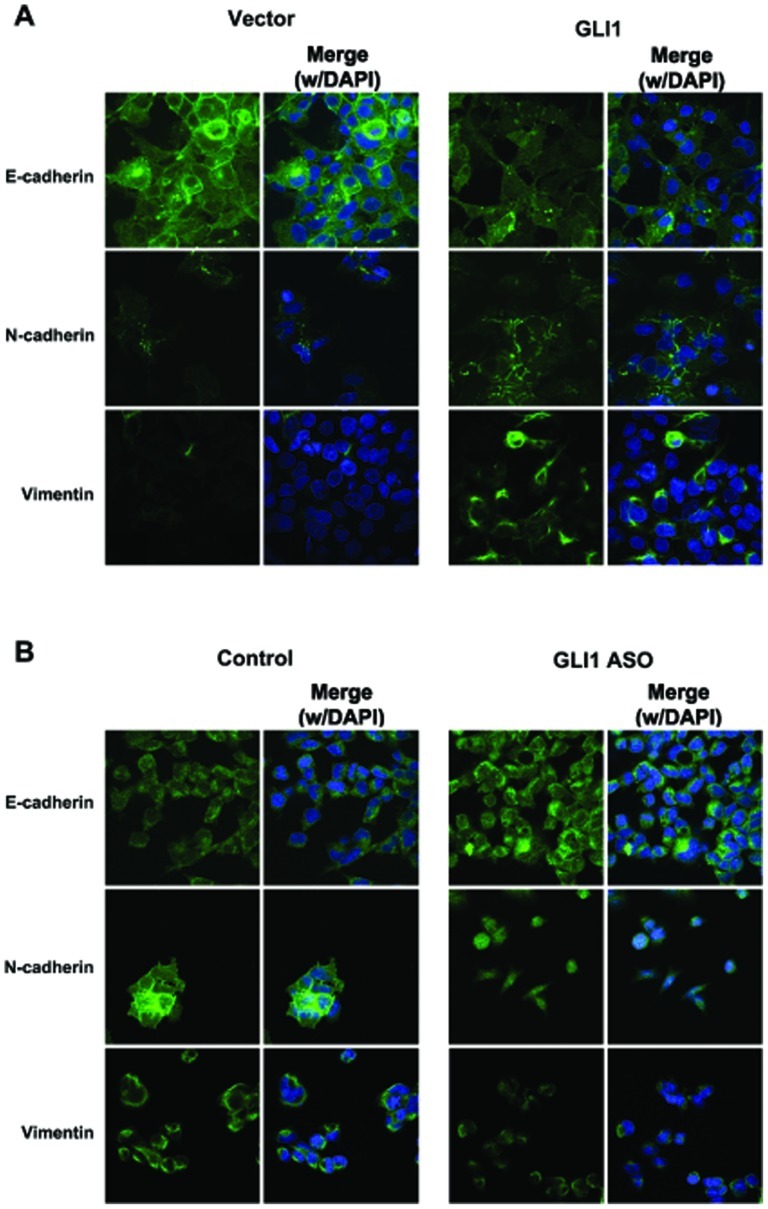
Immunofluorescence studies confirm the role of GLI1 in the regulation of EMT in HCC cells. (A) As assessed by immunofluorescence staining and confocal microscopy, overexpression of GLI1 decreases E-cadherin protein expression and increases the protein expression of both N-cadherin and Vimentin in Huh7 cells. (B) As assessed by immunofluorescence staining and confocal microscopy, knockdown of GLI1 (GLI1 ASO) increases E-cadherin protein expression and decreases the protein expression of both N-cadherin and Vimentin in SNU398 cells. DAPI was used as nuclear staining.

The effect of GLI1 on SNAI1 expression prompted us to examine the effect of GLI1 on EMT by assessing the mRNA and protein expression of the well-established epithelial marker E-cadherin and the mesenchymal markers N-cadherin and Vimentin. E-cadherin mRNA was significantly decreased in Huh7-GLI1 cells compared to Huh7-Vector cells (*P* = 0.002; Figure 4A), in contrast, the mRNA levels of N-cadherin and Vimentin were both significantly up-regulated by overexpression of GLI1 (*P* = 0.002; Figure 4A). Consistent with the mRNA results, overexpression of GLI1 decreased E-cadherin protein expression and increased N-cadherin and Vimentin protein expression as shown by both Western immunoblotting and immunofluorescence (Figure 4B and 5A). Additional experiments performed in cells with endogenous low levels of GLI1: SNU423 and SK-HEP-1, show similar EMT changes upon GLI1 overexpression (Figure S5). On the other hand, knockdown of GLI1 in SNU398 cells up-regulated E-cadherin, and down-regulated both N-cadherin and Vimentin (*P* = 0.004, *P*<0.0001 and *P*<0.0001, respectively; Figure 4A, 4B and 5B) at both the mRNA and protein levels. Similar results were obtained in SNU449 and SNU 475 cells (Figure S5). These results, which show reversal of the EMT phenotype after knockdown of GLI1 in SNU398 cells, are consistent with the observed decreases in cell migration and invasion, two cellular events associated with EMT changes in cancer cells (Figure 1C and 1D).

**Figure 6:**
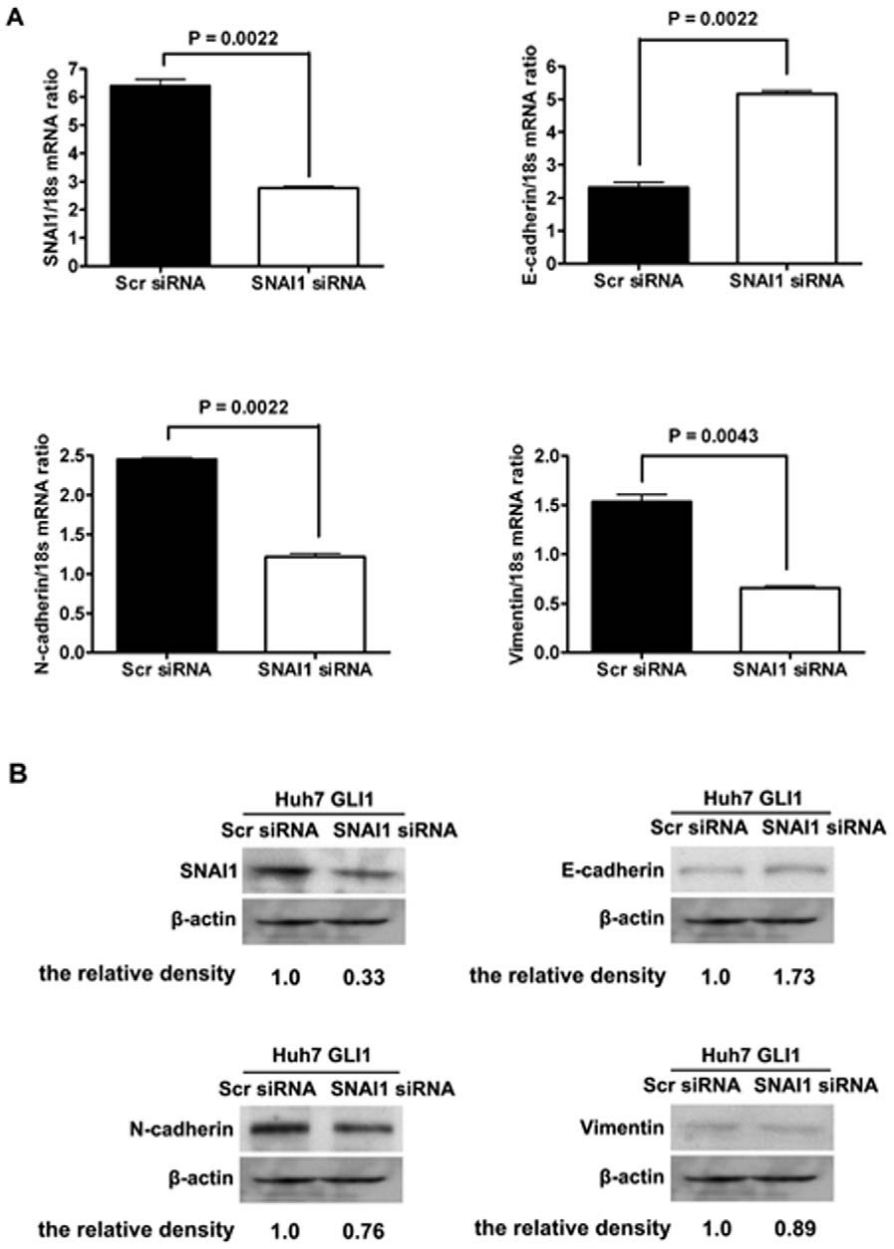
Knockdown of SNAI1 reverses the EMT phenotype induced by GLI1 in HCC cells. (A) SNAI1 siRNA decreases SNAI1 expression in Huh7 cells transfected stably with GLI1 expressing plasmid. SNAI1 siRNA increases E-cadherin expression (B) and decreases the expression of both N-cadherin (C) and Vimentin (D) in Huh7 cells transfected stably with GLI1 expressing plasmid.

To determine whether SNAI1 plays a critical role in the GLI1-induced EMT phenotype in HCC, we silenced SNAI1 expression in Huh7 cells overexpressing GLI1 using siRNA. Knockdown of SNAI1 was confirmed by qRT-PCR and Western immunoblotting (Figure 6A and 6B). The mRNA and protein expression of E-cadherin were both significantly increased in cells transfected with SNAI1 siRNA, while the expression of both N-cadherin and Vimentin were decreased at both the mRNA and protein levels (Figure 6A, and B, lower panel). These data show that knockdown of SNAI1 can inhibit the EMT phenotype induced by GLI1 in HCC, which further supports the hypothesis that SNAI1 could mediate GLI1-induced EMT in HCC.

### The GLI1-SNAI1 axis is required for TGFβ1 EMT induction

**Figure 7:**
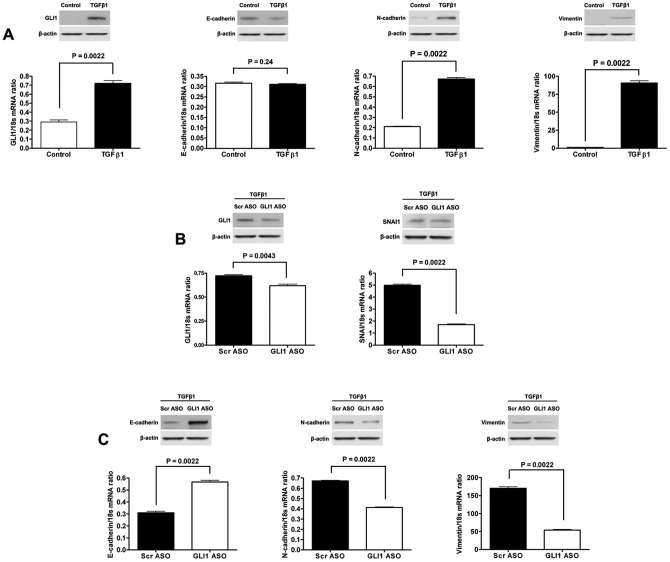
GLI1 is induced by TGFβ1 and mediates the mechanism by which TGFβ1 induces EMT in HCC cells. (A) TGFβ1 increases GLI1 expression in Huh7 cells. And TGFβ1 is also found to decrease E-cadherin expression, while increasing the expression of N-cadherin, Vimentin and SNAI1 in Huh7 cells. (B) Suppressing the TGFβ1 induced up-regulation of GLI1 in Huh7 cells by GLI1 ASO decreased the expression of both GLI1 and SNAI1 in Huh7 cells. (C) Suppressing the TGFβ1 induced up-regulation of GLI1 in Huh7 cells by GLI1 ASO increases E-cadherin expression and decreases the expression of both N-cadherin and Vimentin.

To assess whether this GLI1-SNAI1 axis can function as an effector of pathways that control EMT in HCC cells we examined the effect of knockdown of GLI1 on the EMT induced by TGFβ1, a known regulator of EMT in HCC cells [12], [18]. Treatment of Huh7 cells with TGFβ1 led to up-regulation of GLI1 expression (Figure 7A). Concomitantly, TGFβ1 significantly induced the expression of N-cadherin, Vimentin and SNAI1 (Figure 7A and Figure S5). Although no significant difference was found in E-cadherin expression, the effects on N-cadherin and Vimentin still indicate at least a partial TGFβ1 induction of the EMT phenotype in Huh7 cells. To investigate whether GLI1 is involved in TGFβ1-induced EMT in HCC cells, we transfected Huh7 cells with GLI1 ASO before TGFβ1 treatment. The GLI1 ASO suppressed the TGFβ1-induction of GLI1 expression (Figure 7B). Concomitantly, SNAI1 expression was significantly decreased (Figure 7B). Consistent with these results, transfection of TGFβ1-treated Huh7 cells with GLI1 ASO significantly increased E-cadherin expression and decreased the expression of both N-cadherin and Vimentin (Figure 7C). These data suggest that the GLI1-SNAI1 axis plays an important role in the mechanism by which TGFβ1 induces the EMT in HCC cells and define GLI1 as a central mediator of this cellular process.

## Discussion

The natural history of HCC is characterized by rapidly infiltrating growth and early metastasis. Due to these pathologic features, approximately 60–80% of HCC patients are diagnosed at an advanced stage at which they are not candidates for curative treatments [19], [20]. Achieving improvements in therapy will require a better understanding of the molecular mechanisms regulating HCC invasion and metastasis. The EMT has been shown to contribute to tumor formation and metastasis of HCC, however, the mechanisms by which EMT is regulated in HCC have not been completely elucidated [8], [21]–[24]. We have found that the transcription factor GLI1 expression was positively associated with the EMT phenotype and with intrahepatic metastasis and portal venous invasion of human HCCs [11]. In this study, we show a new analysis of microarray expression data demonstrating that GLI1 overexpression in HCC tissues is associated with more rapid recurrence of HCC tumors after surgery. Corresponding *in vitro* studies in cell line models of overexpression or suppression of GLI1 expression showed that overexpression of GLI1 increased proliferation, viability, migration, invasion and colony formation by HCC cells. Conversely, GLI1 knockdown led to a decrease in these cancer-associated phenotypes in HCC cells. GLI1 induced the EMT in HCC cells. Further mechanistic studies identified SNAI1 as a transcriptional target of GLI1 in HCC cells and showed that this GLI1-SNAI1 axis mediates the TGFβ1-induced EMT in HCC cells. Thus, we have defined a mechanism regulated by GLI1 controlling the EMT in HCC cells. These results are in alignment with previous reports suggesting that GLI1 promotes recurrence and metastasis in other tumor types such as colon cancer [25]. Together, our findings demonstrate a central role for GLI1 in regulation of the EMT in liver cancer and expand the repertoire of pathways that modulate this cellular process.

In most models of epithelial cell carcinogenesis, TGFβ1 inhibits cell proliferation by arresting tumor cells in the G1 phase [26], thus functioning as a tumor suppressor. On the other hand, TGFβ1 has also been shown to promote tumor progression and metastasis of established cancers, in part by the induction of the EMT [18], [27], [28]. This apparent conversion in TGFβ1 function after tumor initiation is known as the “TGFβ1 paradox” [29]–[31]. It is important to understand the downstream effectors of the TGFβ1 signaling pathway and identify the “molecular switch or switches” mediating the “TGFβ1 paradox”. TGFβ1 is considered a major driver of the EMT in HCCs [12], [18], [32]. While investigating the mechanistic interactions between TGFβ1 and GLI1 signaling during induction of the EMT in HCCs, we found that induction of the EMT in Huh7 cells by TGFβ1 was associated with a significant increase in GLI1 expression. When GLI1 expression was silenced, TGFβ1 induction of the EMT phenotype was abrogated and the expression of SNAI1, a known downstream effector of TGFβ1-induced EMT, was also decreased.

SNAI1 is an effector transcription factor that has been shown to induce the EMT through direct suppression of E-cadherin transcription in cancer and thus contribute to tumor cell growth, migration and invasion [33]–[34]. In this study, we found that overexpression of GLI1 up-regulated SNAI1 in Huh7 cells and knockdown of GLI1 down-regulated SNAI1 in SNU398 cells. Further, we also confirmed by ChIP assay that GLI1 directly binds to the SNAI1 promoter in HCC cells. These data show that GLI1 can increase SNAI1 expression in HCC by directly promoting its transcription. Next, by assessing the expression of EMT markers and differences in cell migration and invasion, we found that overexpression of GLI1 induced a significant EMT phenotype in Huh7 cells, while knockdown of GLI1 in SNU398 cells reversed their original mesenchymal-like phenotype. Those results confirm that GLI1 can induce the EMT in HCC. To further investigate whether SNAI1 contributes to the GLI1 induced EMT, we silenced SNAI1 expression in Huh7 cells transfected with a GLI1 expressing plasmid and found that the EMT phenotype was reverse d. These results are in agreement with previous studies demonstrating that GLI1 controls the expression of SNAI1 and uses this transcription factor as a cellular effector to regulate gene expression and transformation [16], [35]–[37]. Reports by Li and colleagues showed that GLI1 requires the induction of SNAI1 to promote transformation in epithelial cells and to regulate the activity Wnt oncogenic signaling [38]. Similarly, Kuphal et al. demonstrate that GLI1 controls cell proliferation via a SNAI1-dependent regulation of CYLD [37]. Together these results strongly suggest that the induction of the EMT by GLI1 in HCC is mediated through SNAI1.

In conclusion, this study shows that GLI1 promotes colony formation, proliferation, viability, migration and invasion of HCC cells, which may contribute to HCC recurrence. Further, we show that GLI1 induces the EMT through direct up-regulation of the EMT-inducer SNAI1. The GLI1-SNAI1 axis also appears to be involved in the mechanism by which TGFβ1 induces the EMT in HCC, which suggests that up-regulation of GLI1 may be a potential “switch” for the “TGFβ1 paradox”. Taken together, our data imply that GLI1 plays an important part in mediating HCC progression and may serve as a therapeutic target for HCC.

## Materials and Methods

### Ethics Statement

The microarray gene expression analysis of human HCCs reported in this study was approved by the Institutional Review Boards of the Mayo Clinic, the Cancer Institute of the Chinese Academy of Medical Sciences, the University of Leuven and the US National Cancer Institute. Deidentified anonymized samples were either from patients who had provided verbal or written informed consent or were surgical waste tissues from deceased patients, which were used with Institutional Review Board approval. Verbal consent was the standard requirement of the Mayo Clinic Institutional Review Board at the time the Mayo Hepatobiliary Neoplasia Biorepository was initiated in 2001. Verbal consent was obtained from patients and documented in the patient medical record as instructed and approved by the Mayo Clinic Institutional Review Board from 2001 to 2003. Beginning in 2003, the Mayo Clinic Institutional Review Board required written informed consent and this was obtained and the signed consent form was scanned into each patient's electronic medical record.

### Cell Culture and Treatment

Ten HCC cell lines (Hep3B, HepG2, PLC/PRF5, SK-HEP-1, SNU398, SNU423, SNU387, SNU475, SNU182 and SNU449) were obtained from the American Type Culture Collection (Manassas, VA) and were cultured as recommended. Huh7 was obtained from the Japan Health Science Research Resources Bank (HSRRB, Osaka, Japan). Normal human hepatocytes were isolated and cultured from a patient without any known liver disease at Mayo Clinic, Rochester, MN. For TGFβ1 treatment, Huh7 cells were incubated with 1% FBS for 24 hours and stimulated with 10 ng/ml of TGF-β1 for 72 hours.

### GLI1-expressing Plasmid Construction and Stable Transfection

The cDNA of GLI1 was cloned into the pCMV-Tag2B vector from Stratagene (Santa Clara, CA). GLI1-negative Huh7 cells were stably transfected with the GLI1-expressing plasmid using FuGENE® 6 Transfection Reagent (Roche, Indianapolis, IN) and designated as Huh7 GLI1-expressing cells (Huh7-GLI1). Empty pCMV-Tag2B vector was also transfected into Huh7 cells and designated as Huh7 vector control cells (Huh7-Vector). Geneticin (G418) from Invitrogen (Carlsbad, CA) at a dose of 600 µg/mL was used to select stably expressing clones.

### GLI1 Antisense Oligonucleotide and SNAI1 siRNA Transfection

GLI1 antisense oligonucleotide (ASO) and negative control ASO were obtained from ISIS Pharmaceuticals (Carlsbad, CA). The details of these ASO have been reported previously [38]. We transfected the GLI1 ASO into SNU398 cells using Lipofectamine 2000 (Invitrogen, Carlsbad, CA) following the manufacturer's instruction and designated the cells as GLI1-knockdown SNU398 cells (SNU398-GLI1 ASO). Similarly, negative control ASO was transfected into SNU398 cells (SNU398-control ASO). SNAI1 siRNA (sc-38398) was obtained from Santa Cruz Bio (Santa Cruz, CA). Using the siPORT™ NeoFX™ Transfection Agent (Applied Biosystems, Carlsbad, CA), we transfected both SNAI1 siRNA and scrambled siRNA into Huh7 cells which had been previously transfected with GLI1-expressing plasmid according to the instruction manual.

### Quantitative Real-time Reverse Transcription Polymerase Chain Reaction (qRT-PCR)

Total RNA was extracted from HCC cell lines using the RNeasy kit (Qiagen, Valencia, CA). cDNA synthesis was performed using the High Capacity cDNA Reverse Transcription Kit (Applied Biosystems, Carlsbad, CA) to transcribe 2 µg of total RNA. QRT-PCR was performed using ABI TaqMan assays in an ABI 7300 system. The following ABI TaqMan probes were used: 18s rRNA, GLI1, SNAI1, E-cadherin, N-cadherin, and Vimentin. The mRNA levels were normalized to 18s rRNA mRNA levels in the same samples. Each measurement was performed 6 times.

### Western Immunoblotting

After selection with G418 for two weeks, whole cell protein lysates were prepared from Huh-GLI1 cells and Huh7-Vector cells. Whole cell protein lysates were prepared from SNU398-GLI1 ASO cells and SNU398-control ASO cells cultured for 3 days after ASO transfection. Thirty micrograms of protein were separated by denaturing gel electrophoresis. After transfer to PVDF membrane, blots were probed overnight with the following anti-human primary antibodies: rabbit polyclonal GLI1 antibody (V812, 1∶1000 dilution, Cell signaling, Danvers, MA), rabbit monoclonal SNAI1 antibody (C15D3, 1∶1000 dilution, Cell signaling, Danvers, MA), mouse monoclonal E-cadherin antibody (610181, 1∶1000 dilution, BD Transduction Laboratory, San Jose, CA), rabbit polyclonal N-cadherin antibody (sc7939, 1∶200 dilution, Santa Cruz Bio, Santa Cruz, CA), rabbit monoclonal Vimentin antibody (EPR3776, 1∶1000, Abcam, Cambridge, MA) and mouse monoclonal β-actin antibody (A-5316, 1∶1000 dilution, Sigma, St. Louis, MO). Blots were then incubated with anti-rabbit (ALI3403, 1∶5000 dilution, Biosource, Carlsbad, CA) or anti-mouse secondary antibodies conjugated with HRP (A3673, 1∶5000 dilution, Sigma, St. Louis, MO), and signals were visualized using the HyGLO HRP detection kit from Denville (Metuchen, NJ). β-actin was measured to control for equal loading.

### Immunofluorescence and Confocal Microscopy

Huh7-GLI1 cells, Huh7-Vector cells, SNU398-GLI1 ASO cells and SNU398-control ASO cells were seeded on Lab-Tek II Chamber Slides (Nalgene Nunc International, Naperville, IL) and incubated for 24 hours. The cells were rinsed with Dulbecco's PBS (D-PBS) at room temperature and fixed for 20 min with 2.5% formaldehyde in PIPES buffer. After rinsing with D-PBS, the cells were incubated in blocking buffer (5% normal goat serum and 5% glycerol in D-PBS) for 1 h at 37°C. The cells were then incubated with primary antibodies against GLI1, SNAI1, E-cadherin, N-cadherin and Vimentin overnight at 4°C, rinsed 3 times, 10 min each time, with D-PBS, and incubated with the appropriate FITC-labeled secondary antibodies (Alexa Fluor 488, 1∶1000 dilution, Invitrogen, Carlsbad, CA) for 2 h at room temperature. Cells were then washed three times for 5 min each time with D-PBS, rinsed briefly with distilled water, mounted with DAPI on glass slides, and examined by confocal microscopy.

### Luciferase Reporter Assay

GLI-Luciferase reporter plasmid containing eight consecutive GLI-binding sites downstream of the luciferase gene (8XGLI) was kindly provided by Dr. Chi-chung Hui (Research Institute, Toronto, Ontario, Canada) [39]. The SNAI1 promoter wild type and mutant promoters were kindly provided by Dr. Cynthia Wetmore (Department of Oncology, St. Jude Children's Research Hospital, Memphis, TN) [17]. Cells plated in six-well plates at 60% confluency were transfected with the reporter plasmid using FuGENE® 6 Transfection Reagent. Luciferase activity was measured using the Dual-Luciferase Reporter assay from Promega (Madison, WI) and normalized by protein quantification. Each data point represents an average of six independent transfections.

### Cell Proliferation and Viability Assays

After selection with G418 for two weeks or transient transfection, HCC cells were used in cell proliferation and cell viability assays, respectively. For the proliferation assay, HCC cells were seeded into 96-well plates at 5000 cells per well for 24 hours and assessed using the BrdU ELISA kit (Roche, Indianapolis, IN). Soft agar colony formation assays were performed in 96-well plates using the CytoSelect™ 96-Well Cell Transformation Assay kit (Cell Biolabs, San Diego, CA). Briefly, 5000 cells were seeded per well in soft agar. After culturing cells for 6 days, we removed the culture medium completely, solubilized the soft agar, lysed the cells and added the CyQuant working solution. The results were read with on a fluorescence plate reader using a 485/520 nm filter set. The 3-(4, 5-dimethylthiazol-2-yl)-2,5-diphenyl tetrazolium bromide (MTT) assay was used to assess cell viability at 24, 48, 72 and 96 hours. The experiments were performed with at least six replicates.

### Migration Assay

HCC cells were plated onto 6-well plates and grown to confluency. Scratch wounds were made with a 1000-µL-pipette tip. The wounds were photographed with a phase-contrast microscope at 0, 24 and 48 hours. Cell migration was quantitated by measuring the width of the wounds. The experiments were performed with at least six replicates.

### Invasion Assay

We used the QCM™ 96-well cell invasion assay kit (Millipore, Billerica, MA) for the invasion assays. HCC cells were first incubated in serum-free medium for 24 hours. They were then seeded into the invasion chamber at 1×10^5^ cells per well in serum-free medium. Medium with 10% fetal bovine serum (FBS) was added into the lower chamber. After incubation for 24 hours at 37°C in a CO2 incubator (5% CO_2_), invaded HCC cells were dislodged completely from underneath the invasion chamber using the cell detachment solution and added to the lysis buffer/dye solution mixture. The results were read with fluorescence plate reader using a 480/520 nm filter set. The experiments were performed with at least six replicates.

### Chromatin Immunoprecipitation Assay

Cells were fixed with 1% formaldehyde for 10 minutes at room temperature and were processed further for immunoprecipitation of chromatin. Chromatin immunoprecipitation (ChIP) was carried out with the EZ-Magna ChIP™ Chromatin Immunoprecipitation Kit (Millipore, Billerica, MA). Briefly, associated proteins were cross-linked with 1% formaldehyde followed by cell lysis and DNA shearing via 15 cycles of sonication. Cells were then immunoprecipitated using a GLI1 antibody (Cell Signaling Technology, Danvers, MA), or normal rabbit IgG (Santa Cruz, CA). After that, cross-links were removed and the immunoprecipitated DNA was purified and subsequently amplified by PCR. The primers used to detect the SNAI1 promoter are designed to amplify a region spanning putative GLI binding elements identified within the human SNAI1 promoter. The sequences are Forward: 5′- gtcttccttcacaggtggaaac-3′ and Reverse: 5′- cgtgaagatcctcgatcctgtg-3′ producing an approximately 200 bp product. PCR products were separated by electrophoresis and then visualized on a 2% agarose gel.

### Microarray Gene Expression Profiling

HCC tumor and adjacent benign tissues were obtained from 139 individuals undergoing surgical resection for HCC at centers in Asia, Europe, and the United States. The study was approved by the Institutional Review Boards of the Mayo Clinic, the Cancer Institute of the Chinese Academy of Medical Sciences, the University of Leuven and the US National Cancer Institute. The demographic information, clinical features and follow-up information on the patients have been reported previously [32]. These HCC samples are different from those in our other previous reports [11]. The details of microarray gene expression profiling have also been described in the previous paper [40]. The data have been deposited at Gene Expression Omnibus (GEO/GPL1528, GEO/GPL1529, GEO/GSE1897, GEO/GSE1898 and GEO/GSE4024). Briefly, total RNAs were isolated from frozen liver tissue using CsCl density-gradient centrifugation. 20 µg of total RNA from tissues was used to derive fluorescently (Cy-5 or Cy-3) labeled cDNA. We carried out at least two hybridizations for each tissue using a dye-swap strategy to eliminate dye-labeling bias. Specimens were analyzed at the US National Cancer Institute using the Human Array-Ready Oligo Set (Version 2.0) containing 70-mer probes of 21,329 genes from Qiagen (Valencia, CA).

### Statistical Analysis

All data are expressed as means and standard errors of the mean. Differences between groups were compared with the Mann-Whitney test. Differences between the Kaplan-Meier curves of HCC patients with high and low/no-GLI1 expression in the HCC tissue in comparison with the adjacent benign tissue were analyzed using the log rank test.

## Supporting Information

Figure S1 **GLI1 is differentially regulated in HCC cell lines.** Five of the tested HCC cell lines (PLC/PRF5, SNU182, SNU398, SNU449, and SNU475) express GLI1 mRNA at a higher level than normal human hepatocytes. SNU398 is the highest expressing cell line and expresses GLI1 mRNA at over 55-times the level in normal hepatocytes. Other HCC cell lines including Huh7, SK-HEP-1, Hep3B, SNU387, SNU423 and the hepatoblastoma cell line HepG2 express lower GLI1 mRNA levels than normal human hepatocytes.
(TIF)

Figure S2 **GLI1 protein expression in HCC cell lines.** Western blotting confirms that GLI1 protein expression correlates with GLI1 mRNA in HCC cells. Tubulin was used as a housekeeping control.
(TIF)

Figure S3 **GLI1 signaling can be efficiently modulated in HCC cells.** (A) At the levels of both mRNA and protein, GLI1 expression is increased by GLI1 expressing plasmid in Huh7 cells and decreased by GLI1 ASO in SNU398 cells. (B) GLI reporter luciferase activity is increased by GLI1 expressing plasmid in Huh7 cells and decreased by GLI1 ASO in SNU398 cells.
(TIF)

Figure S4 **SNAI1 is a transcriptional target of GLI1 in HCC cells.** (A) As measured by qRT-PCR, SNAI1 expression is decreased by knockdown of GLI1 in SNU449 and SNU475 cells (B) qRT-PCR confirm the efficiency of GLI1 overexpression and knockdown in the indicated HCC lines.
(TIF)

Figure S5 **GLI1 induces the EMT in HCC ells.** (A) qRT-PCR showing that TGFβ1 induces the expression of SNAI1 in SNU398. (B-C) As assessed by qRT-PCR, overexpression of GLI1 decreases E-cadherin mRNA expression in SNU423 cells (B) and increases the mRNA expression of Vimentin in SK-HEP-1 cells (C), while knockdown of GLI1 increases E-cadherin mRNA expression and decreases the mRNA expression of both N-cadherin and Vimentin in SNU475 cells. (D) mRNA expression in SNU475 shows that knockdown of GLI1 increase the expression of E-cadherin levels and dimished the expression of N-Cadherin and Vimentin.
(TIF)

Table S1 **Demographic information and clinical features of patients from GLI1 high expresser group and GLI1 low/non-expresser group.**
(DOC)

Table S2 **Demographic information and clinical features of patients from high GLI1 group and low GLI1 group.**
(DOC)
