# Supplementary figures and images for: The Transcription Factor GLI1 Mediates TGFβ1 Driven EMT in Hepatocellular Carcinoma via a SNAI1-Dependent Mechanism
Source: PLoS One. 2012 Nov 19;7(11):e49581. doi: 10.1371/journal.pone.0049581 (PMC3501480; doi:10.1371/journal.pone.0049581)

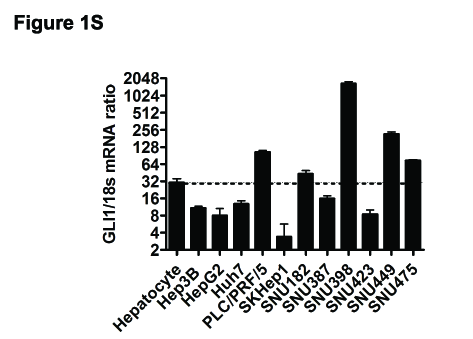

Supplement: Figure S1 — GLI1 is differentially regulated in HCC cell lines. Five of the tested HCC cell lines (PLC/PRF5, SNU182, SNU398, SNU449, and SNU475) express GLI1 mRNA at a higher level than normal human hepatocytes. SNU398 is the highest expressing cell line and expresses GLI1 mRNA at over 55-times the level in normal hepatocytes. Other HCC cell lines including Huh7, SK-HEP-1, Hep3B, SNU387, SNU423 and the hepatoblastoma cell line HepG2 express lower GLI1 mRNA levels than normal human hepatocytes. (TIF) [file pone.0049581.s001.tif]

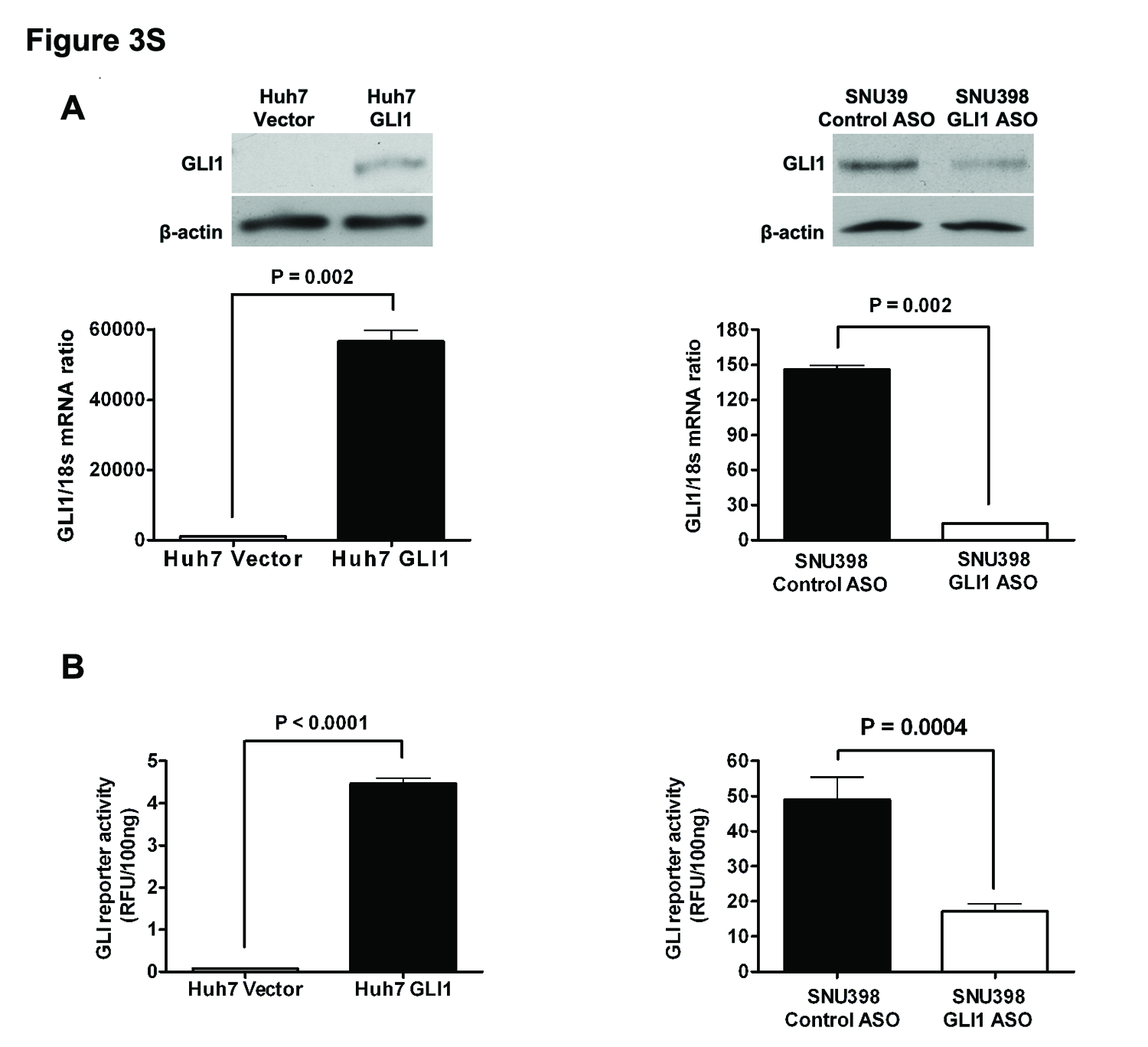

Supplement: Figure S3 — GLI1 signaling can be efficiently modulated in HCC cells. (A) At the levels of both mRNA and protein, GLI1 expression is increased by GLI1 expressing plasmid in Huh7 cells and decreased by GLI1 ASO in SNU398 cells. (B) GLI reporter luciferase activity is increased by GLI1 expressing plasmid in Huh7 cells and decreased by GLI1 ASO in SNU398 cells. (TIF) [file pone.0049581.s003.tif]

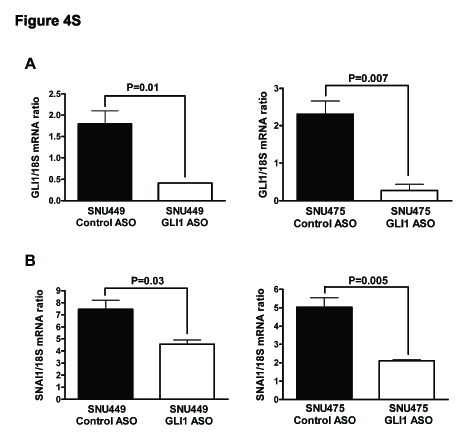

Supplement: Figure S4 — SNAI1 is a transcriptional target of GLI1 in HCC cells. (A) As measured by qRT-PCR, SNAI1 expression is decreased by knockdown of GLI1 in SNU449 and SNU475 cells (B) qRT-PCR confirm the efficiency of GLI1 overexpression and knockdown in the indicated HCC lines. (TIF) [file pone.0049581.s004.tif]

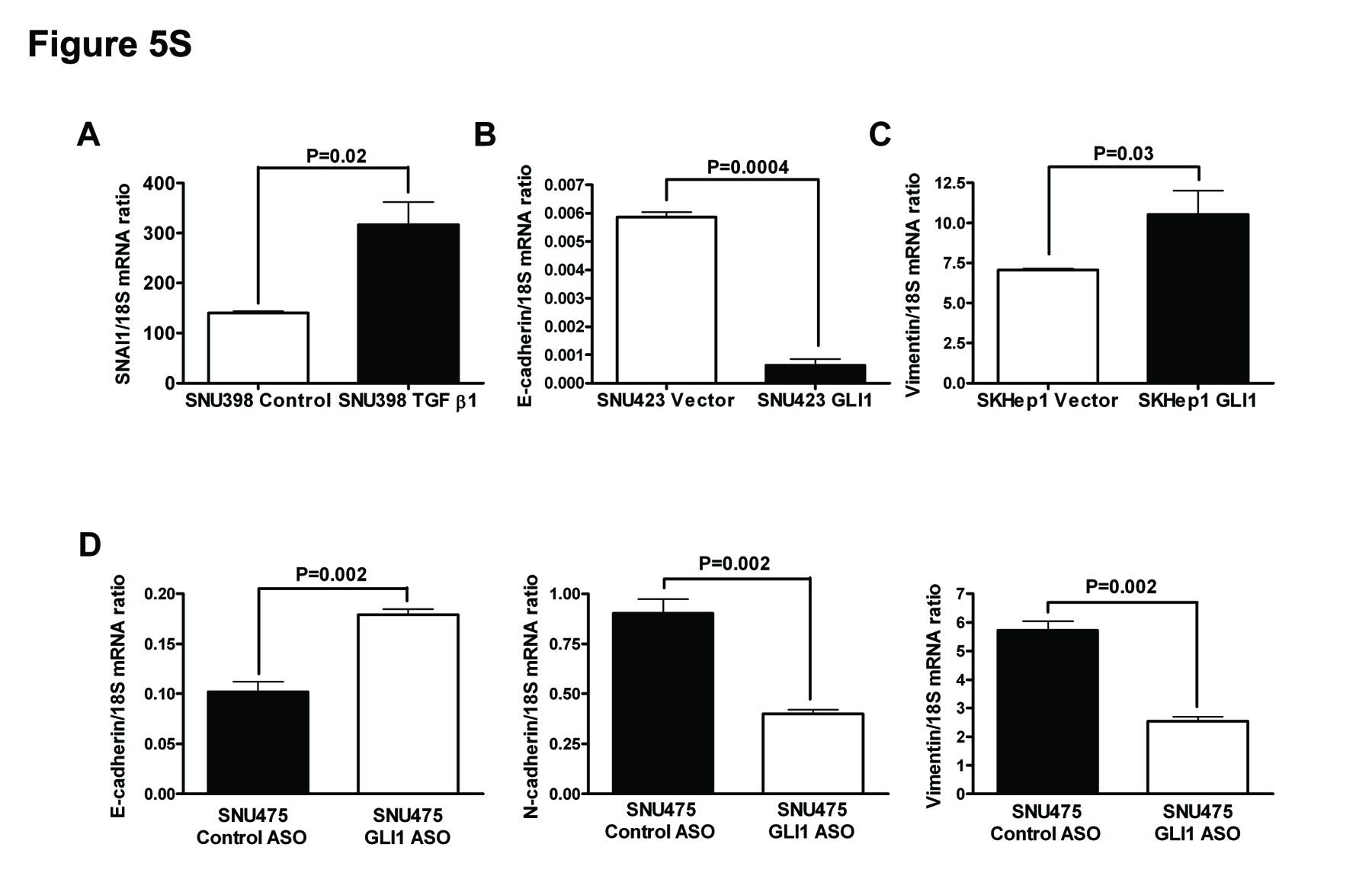

Supplement: Figure S5 — GLI1 induces the EMT in HCC ells. (A) qRT-PCR showing that TGFβ1 induces the expression of SNAI1 in SNU398. (B-C) As assessed by qRT-PCR, overexpression of GLI1 decreases E-cadherin mRNA expression in SNU423 cells (B) and increases the mRNA expression of Vimentin in SK-HEP-1 cells (C), while knockdown of GLI1 increases E-cadherin mRNA expression and decreases the mRNA expression of both N-cadherin and Vimentin in SNU475 cells. (D) mRNA expression in SNU475 shows that knockdown of GLI1 increase the expression of E-cadherin levels and dimished the expression of N-Cadherin and Vimentin. (TIF) [file pone.0049581.s005.tif]
