# Supplementary material for: The Transcription Factor GLI1 Mediates TGFβ1 Driven EMT in Hepatocellular Carcinoma via a SNAI1-Dependent Mechanism
Source: PLoS One. 2012 Nov 19;7(11):e49581. doi: 10.1371/journal.pone.0049581 (PMC3501480; doi:10.1371/journal.pone.0049581)
Supplement: Table S1 — Demographic information and clinical features of patients from GLI1 high expresser group and GLI1 low/non-expresser group. (DOC) [file pone.0049581.s006.doc]

Table 1. Demographic information and clinical features of patients from GLI1 high expresser group and GLI1 low/non-expresser group

|  | | GLI1 high  expressers | GLI1 low/non  expressers | P value |
| --- | --- | --- | --- | --- |
| No. of Patients | | 40 | 99 |  |
| Age (y) (median (range)) | | 62 (9-79) | 56 (26-85) | 0.36 |
| Gender (Male) | | 32 (80.0%) | 70 (70.7%) | 0.18 |
| Etiology | |  |  |  |
| HBV | | 15 (37.5%) | 46 (46.5%) | 0.44 |
| HCV | | 7 (17.5%) | 14 (14.1%) | 0.81 |
| Alcoholic Liver Disease | | 6 (15%) | 13 (13.1%) | 0.99 |
| NASH or NAFLD | | 1 (2.5%) | 2 (2.0%) | 0.86 |
| Hemochromatosis | | 3 (7.5%) | 4 (4.0%) | 0.68 |
| Cryptogenic Cirrhosis | | 1 (2.5%) | 4 (4.0%) | 0.66 |
| Other | | 1 (2.5%) | 3 (3.0%) |  |
| Unknown | | 5 (12.5%) | 9 (81.8%) |  |
| Cirrhosis | | 17 (42.5%) | 52 (52.5%) | 0.28 |
| AFP (ng/mL) | > 300 | 14 (35.0%) | 41 (41.4%) |  |
|  | < 300 | 22 (55.0%) | 51 (51.5%) |  |
|  | NA* | 4 (10.0%) | 7 (7.1%) |  |
| Tumor size (cm) (mean ± SD) | | 6.3 ± 0.5 | 6.7 ± 0.4 | 0.52 |
| Edmonson grade | Grade 1 | 0 (0.0%) | 2 (2.0%) |  |
|  | Grade 2 | 16 (40.0%) | 41 (41.5%) |  |
|  | Grade 3 | 22 (55.0%) | 52 (52.5) |  |
|  | Grade 4 | 2 (5.0%) | 4 (4.0%) |  |
| Vasculature invasion | | 9 (22.5%) | 10 (10.1%) | 0.05 |

* NA: Not Available
