# Supplementary material for: The Transcription Factor GLI1 Mediates TGFβ1 Driven EMT in Hepatocellular Carcinoma via a SNAI1-Dependent Mechanism
Source: PLoS One. 2012 Nov 19;7(11):e49581. doi: 10.1371/journal.pone.0049581 (PMC3501480; doi:10.1371/journal.pone.0049581)
Supplement: Table S2 — Demographic information and clinical features of patients from high GLI1 group and low GLI1 group. (DOC) [file pone.0049581.s007.doc]

Table 2. Demographic information and clinical features of patients from high GLI1 group and low GLI1 group

|  | | | high GLI1 | Low GLI1 | P value |
| --- | --- | --- | --- | --- | --- |
| No. of Patients | | | 35 | 35 |  |
| Age (y) (median (range)) | | | 62 (42-79) | 56 (19-74) | 0.36 |
| Gender (Male) | | | 27 (77.1%) | 28 (80%) | 0.77 |
| Etiology | | |  |  |  |
| HBV | | | 14 (40%) | 21 (60%) | 0.44 |
| HCV | | | 7 (20%) | 3 (8.6%) | 0.31 |
| Alcoholic Liver Disease | | | 6 (17.1%) | 1 (2.9%) | <0.05 |
| NASH or NAFLD | | | 1 (2.9%) | 1 (2.9%) | 1.00 |
| Hemochromatosis | | | 2 (5.7%) | 3 (8.6%) | 0.68 |
| Cryptogenic Cirrhosis | | | 1 (2.5%) | 4 (4.0%) | 0.64 |
| Other | | | 0 (0.0%) | 2 (5.7%) |  |
| Unknown | | | 10 (28.6%) | 6 (17.1%) |  |
| Cirrhosis | | | 15 (42.9%) | 12 (34.3%) | 0.62 |
| AFP (ng/mL) | > 300 | | 12 (34.3%) | 17 (48.6%) |  |
|  | < 300 | | 20 (57.1%) | 17 (48.6%) |  |
|  | NA* | | 3 (8.6%) | 1 (2.8%) |  |
| Tumor size (cm) (mean ± SD) | | | 6.2 ± 0.6 | 7.3 ± 0.7 | 0.26 |
| Edmonson grade | | Grade 1 | 0 (0.0%) | 0 (0.0%) |  |
|  |  | Grade 2 | 15 (42.9%) | 13 (37.1%) |  |
|  |  | Grade 3 | 18 (51.4%) | 22 (62.9%) |  |
|  |  | Grade 4 | 2 (5.7%) | 0 (0.0%) |  |
| Vasculature invasion | | | 8 (22.9%) | 5 (14.3%) | 0.18 |

* NA: Not Available
